# Rare cause of intestinal obstruction, *Ascaris lumbricoides* infestation: two case reports

**DOI:** 10.4076/1757-1626-2-7970

**Published:** 2009-06-17

**Authors:** Ibrahim Yetim, Orhan Veli Ozkan, Ersan Semerci, Recep Abanoz

**Affiliations:** 1 Department of General Surgery, Faculty of Medicine, Mustafa Kemal UniversityHatay/Turkey; 2 Department of Radiology, Bafra State HospitalSamsun/Turkey

## Abstract

*Ascaris lumbricoides* is common resident of intestine especially low socioeconomic areas in the world. Complication of *Ascaris lumbricoides* has been reported include obstruction of the small intestine, intestinal volvulus and intussusception. We report two children with severe sequelae of intestinal obstruction.

## Introduction

*Ascaris lumbricoides* (AL) is the most common helminth affecting humans and causing important medical and social problems especially in the under-developing countries [1,2]. AL infestation occurs in all age groups but more common in children of preschool age [3]. Obstruction of intestinal tract by a mass of AL is one of the serious and lethal complications. Early diagnosis of obstruction by ultrasonography (USG) is likely possible [1,4]. We presented two cases with intestinal obstruction induced by AL.

## Case presentation

### Case 1

**Figure 1.**
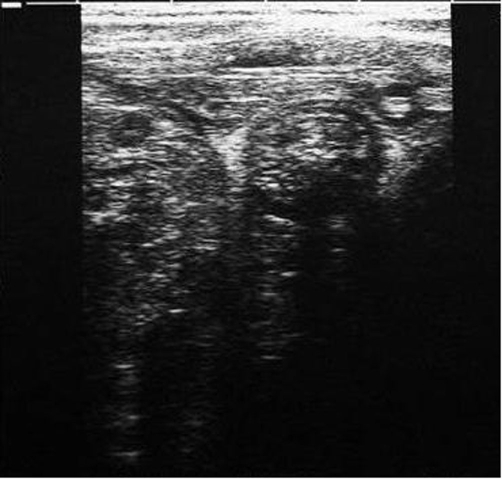
‘Railway track’ and ‘bull’s eye’ signs are on USG.

A 4-year-old Caucasian male child of Turkish nationality was admitted to the emergency department with abdominal pain and biliary vomiting for three days. Physical examination revealed abdomen tenderness and rigidity. X-ray showed air-fluid levels indicative of intestinal obstruction. USG demonstrated masses in the intestinal lumen. Parallel paired lines like ‘railway track’ and ‘bull’s eye’ sign were seen on USG (Figure 1). At laparotomy, he had necrosis of ileal part of approximately 20 cm. The necrosis parts were resected and evacuated the two Ascaris masses. Primary end-to-end anastomosis was performed. Patient was discharged 7^th^ day postoperatively without complication.

### Case 2

**Figure 2.**
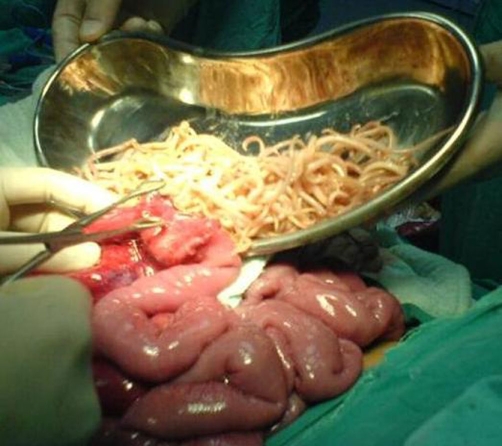
Extraction of *Ascaris lumbricoides* from jejunum via enterotomy.

A 7-year-old Caucasian boy of Turkish nationality was admitted to the emergency department with abdominal pain and vomiting for three days. His x-ray and sonographic features were similar to the first patient. At laparotomy (Figure 2), he had jejunal masses milking by enterotomies and sutured and closed primarily. There was no complication postoperatively and he was discharged 5^th^.

## Discussion

AL is the facultative parasite and resides in human intestinal tract as a harmless inhabitant through its adult life. Ascaris infestation affects especially children reside in socioeconomic areas and with malnutrition and immune deficiencies [5]. They live from stomach to ileocecal valve without causing any serious symptoms. When environment may become change to intolerable for their living, they migrate to more appropriate areas of intestinal tract. AL may cause serious problems at this migration including pancreatitis, cholecystitis, liver abscess, intestinal obstruction and even perforation [6,7]. Diagnosis with clinical symptoms and hematological investigation frequently is not possible. X-ray may show air fluid levels. USG may show two pairs echogenic tubular structures (railway track) longitudinally and bull’s eye horizontally [1,8]. Tubular structures may have active movements that could make diagnosis easily. USG is a simple and reliable method for diagnosis of AL obstruction [6,9], as seen in our both of cases.

The most common acute complication of AL is intestinal obstruction. The rate of mortality from intestinal obstruction is 5.7% below the age of 10 years [4]. Partial intestinal obstruction from AL may resolve spontaneously with the conservative treatment including bowel rest, intravenous fluids, and nasogastric decompressing [10]. When mechanical obstruction persists, bolus of worm acts a fixed point, and leads to intussusception or volvulus. Ascaris may also excrete neurotoxins and anaphylatoxins leading to small bowel spasticity and inflammation. These toxins may induce the mechanical obstruction as well [5,11]. Volvulus, intussusception or increasing pressure to the intestinal wall causes necrosis [12]. In case of necrosis, resection and primary anastomosis are necessary. Piperazine citrate is useful postoperatively.

In conclusion, AL should be kept in mind in preschool children with sudden-acute intestinal obstruction. USG is a very useful tool for its diagnosis.

## List of abbreviations

AL: *Ascaris lumbricoides*
USG: Ultrasonography
